# Adjunct Corticosteroids in Children With Community-Acquired Pneumonia Presenting to the Emergency Department: A Systematic Review and Meta-Analysis

**DOI:** 10.7759/cureus.114926

**Published:** 2026-08-21

**Authors:** Sarra Elnour Ahmed Elnour, Meshari Dalbouh, Hana Gasmelsaid Almamoun Gamaraldin, Alaa Hamed Hag Elzain Eltoum, Muna Omer Abdelgayoum Ahmed, Tyseer Hassan Abdelgadie Abdelhafeez, Hind GasmElseed

**Affiliations:** 1 Pediatric Emergency Medicine, Armed Forces Hospital Southern Region, Ministry of Defense Health Services, Khamis Masheat, SAU; 2 Pediatric Emergency Medicine, Ministry of Health, King Faisal Medical City for Southern Regions-Asir Cluster, Abha, SAU; 3 Pediatrics, Sarat Ebida General Hospital, Sarat Ebida, SAU; 4 Pediatrics, Tipperary University Hospital, Tipperary, IRL; 5 Pediatrics, University Hospital Kerry, Kerry, IRL; 6 Emergency Medicine, Dr. Soliman Fakeeh Medical Center, Makkah, SAU; 7 Pediatrics, Dubai Health Authority, Ajman, ARE

**Keywords:** children, community-acquired pneumonia, corticosteroids, dexamethasone, emergency department, meta-analysis, methylprednisolone, systematic review

## Abstract

Community-acquired pneumonia (CAP) is a leading reason for acute pediatric presentation, and the exuberant host inflammatory response that characterizes severe disease has prompted interest in adjunct systemic corticosteroids. We systematically reviewed and, where feasible, meta-analyzed the randomized evidence in children.

Following PRISMA 2020, we searched PubMed, the Excerpta Medica database (Embase), the Cochrane Library, Web of Science, and ClinicalTrials.gov for randomized controlled trials comparing adjunct systemic corticosteroids plus standard care against standard care or placebo in children (<18 years) with CAP. Risk of bias was assessed with the Cochrane Risk of Bias 2 (RoB 2) tool for randomized trials. Outcomes reported by more than one trial were pooled with random-effects models, with sensitivity analyses across alternative variance estimators; medians were converted to means using an established conversion method. Trial-specific primary outcomes were synthesized narratively.

Three trials (251 children) were included. Each reported a significant benefit on its own primary outcome: shorter fever duration and fewer complications with methylprednisolone in severe CAP; shorter time to recovery with dexamethasone in parapneumonic effusion; and lower early treatment failure with dexamethasone in severe CAP. Pooled length of hospital stay favored corticosteroids (mean difference −2.10 days, 95% CI −6.79 to 2.59), with substantial heterogeneity reflecting differing clinical settings (I² = 91%). All-cause mortality did not differ between groups (risk ratio 0.74, 95% CI 0.14 to 3.85). Corticosteroids were well tolerated, with transient hyperglycemia the most common adverse effect.

Across three small, clinically heterogeneous randomized trials, adjunct systemic corticosteroids improved each trial's own primary outcome and were well tolerated. Because the trials were few, diverse in population and regimen, and imprecise when pooled, the current evidence is preliminary and hypothesis-generating and is insufficient to support routine use. The consistent, favorable signal supports adequately powered, phenotype-enriched pediatric trials with harmonized outcomes.

## Introduction and background

Community-acquired pneumonia (CAP) remains one of the most common serious infections of childhood and a leading reason for acute presentation to the emergency department (ED) and for subsequent hospitalization worldwide [1]. Although conjugate pneumococcal and *Haemophilus influenzae* type b vaccines have reduced severe bacterial disease, *Streptococcus pneumoniae* continues to impose a substantial global burden of childhood pneumonia morbidity and mortality [2]. The clinical trajectory of CAP is shaped not only by microbial invasion but also by the host inflammatory response: a controlled response is required for pathogen clearance, whereas an exuberant or dysregulated response can propagate alveolar injury, impair gas exchange, and prolong recovery. This dual nature of inflammation provides the biological rationale for adjunctive immunomodulatory therapy, and it is against this background that systemic corticosteroids, agents that attenuate cytokine-driven inflammation, have been proposed as an adjunct to antibiotics in the acute management of CAP [1].

In adults, corticosteroids have been studied extensively as adjunctive therapy for CAP. A landmark double-blind trial found that dexamethasone shortened the length of hospital stay [3], and a large multicenter trial reported that adjunct prednisone shortened the time to clinical stability, albeit with more hyperglycemia [4]. Contemporary meta-analyses indicate that corticosteroids probably reduce the length of hospital and intensive-care stay and, in severe CAP, may lower short-term mortality, although the magnitude of benefit varies with pneumonia severity, corticosteroid type, and treatment duration [5]. In severe CAP with a heightened inflammatory response, methylprednisolone reduced treatment failure [6], and early hydrocortisone reduced 28-day mortality among adults admitted to intensive care with severe CAP [7]. These findings have prompted the incorporation of corticosteroids into adult severe-CAP guidance. The pathophysiology, microbial spectrum, and outcome profile of pediatric CAP, nevertheless, differ from those of adults. Mortality is far lower, and the endpoints of greatest relevance are functional (time to clinical recovery, duration of fever and oxygen requirement, and length of stay) rather than survival, so the pediatric question warrants dedicated evaluation.

The pediatric evidence base is comparatively focused. Observational analyses have reached differing conclusions: retrospective multicenter data in hospitalized children have linked adjunct corticosteroids to shorter stays in some subgroups but to longer stays in children without concomitant reactive-airway features, raising the possibility that part of the observed benefit reflects co-treatment of wheezing illness [8]. In the outpatient setting, corticosteroid co-prescription has not been shown to reduce treatment failure [9]. A small number of randomized controlled trials have directly evaluated systemic corticosteroids in children with CAP, and they differ in the corticosteroid used, the severity and phenotype of pneumonia enrolled, and the outcomes measured.

The emergency department is the point of first medical contact for most children with acute CAP and the setting in which the decision to initiate adjunctive therapy is typically made, which motivates a focus on this early decision point. However, since no pediatric randomized trial has exclusively recruited participants from the emergency department, the eligible trials included children who were assessed and managed through both acute-care and inpatient pathways; therefore, the present findings apply to the broader acute-care pediatric CAP population that presents through or is triaged from the emergency department. We conducted a systematic review and meta-analysis of randomized controlled trials to evaluate the efficacy and safety of adjunct systemic corticosteroids, compared with standard antibiotic therapy alone or placebo, in children with CAP requiring acute or emergency assessment.

## Review

Materials and methods

Study Design

This systematic review and meta-analysis was designed, conducted, and reported in accordance with the PRISMA 2020 statement [10]. The review was not prospectively registered in a public registry; the eligibility criteria, primary and secondary outcomes, and analytic methods were, however, specified a priori before study selection and data extraction.

Eligibility Criteria

We included randomized controlled trials enrolling children (<18 years) with CAP presenting for acute care that compared adjunct systemic corticosteroids (oral or intravenous) plus standard care against standard care alone or placebo and reported at least one prespecified clinical outcome. Because no existing pediatric randomized controlled trial recruited exclusively from the emergency department, presentation to the emergency department was operationalized as acute presentation of CAP requiring emergency or inpatient assessment rather than as a recruitment-site restriction. Non-randomized and observational studies, reviews, editorials, case reports, conference abstracts, preprints, and studies restricted to *Mycoplasma pneumoniae*-refractory pneumonia, inhaled or intrapleural corticosteroids, or non-CAP lower-respiratory infection were excluded. The eligibility framework is summarized in Table 1 using the Population, Intervention, Comparison, Outcome, and Study design (PICOS) structure.

**Table 1:** Eligibility criteria (PICOS framework) PICOS: Population, Intervention, Comparison, Outcome, and Study design; CAP: community-acquired pneumonia; LRTI: lower respiratory tract infection.

Domain	Inclusion	Exclusion
Population	Children <18 years with CAP presenting for acute care	Adults; hospital-acquired/ventilator-associated pneumonia; Mycoplasma-refractory pneumonia; non-CAP LRTI
Intervention	Adjunct systemic (oral/IV) corticosteroid + standard antibiotic care	Inhaled or intrapleural corticosteroids; corticosteroid without standard care
Comparator	Standard antibiotic care alone or placebo	No comparator group
Outcomes	≥1 clinical outcome (length of stay, time to recovery, fever duration, treatment failure, adverse events)	No extractable clinical outcome
Study design	Randomized controlled trial (any language)	Observational studies, reviews, editorials, case reports, abstracts, preprints

Information Sources and Search

We searched five databases-PubMed, Excerpta Medica database (Embase), the Cochrane Library, Web of Science, and ClinicalTrials.gov-from database inception to the final search date (Appendix A), supplemented by hand-searching the reference lists of relevant reviews and included trials. No language restriction was applied. The search combined controlled vocabulary and free-text terms for community-acquired pneumonia, corticosteroids (dexamethasone, methylprednisolone, prednisolone, hydrocortisone, glucocorticoid), and pediatric age filters; the complete search strategy for each database is provided in Appendix A. Records were imported into a reference-management program and de-duplicated automatically, with any residual duplicates removed manually.

Study Selection and Data Extraction

Records were screened by title and abstract, then by full text, in duplicate, with disagreements resolved by discussion. Records clearly ineligible on title and abstract (reviews and editorials, or adult populations) were excluded at the screening stage, and full texts were sought for all potentially eligible reports. Three reports could not be retrieved because the full text was unavailable through institutional subscriptions or interlibrary channels; these reports are listed in Appendix B and were not treated as an eligibility exclusion. Retrieved reports were then assessed against the full eligibility criteria. A standardized form captured study identifiers, design and blinding, sample size per arm, participant age and pneumonia phenotype, corticosteroid agent/dose/route/duration, comparator, co-administered antibiotics, and all reported outcomes with dispersion. Where continuous outcomes were reported as median and interquartile range, means and standard deviations were estimated using the method of Wan et al. [11].

Risk of Bias

Two reviewers independently assessed each trial with the Cochrane Risk of Bias 2 (RoB 2) tool across its five domains, with an overall judgment of low risk, some concerns, or high risk [12]; disagreements were resolved by consensus.

Statistical Analysis

Outcomes reported by more than one trial were pooled using a random-effects model, given the anticipated clinical and methodological diversity of the trials. Between-study variance was estimated with the DerSimonian-Laird method, chosen as the conventional and most widely used estimator to preserve comparability with the existing corticosteroid meta-analysis literature. Because random-effects intervals can be too narrow when few studies are available, we performed sensitivity analyses using alternative between-study variance estimators (restricted maximum likelihood and Paule-Mandel) and applied the Hartung-Knapp adjustment to the primary pooled estimate. Continuous outcomes were summarized as mean differences and dichotomous outcomes as risk ratios, each with 95% CIs. Heterogeneity was quantified with the I² statistic, τ², and Cochran's Q, and interpreted using the Cochrane thresholds (I² of 0%-40% possibly unimportant, 30%-60% moderate, 50-90% substantial, and 75%-100% considerable). The median-to-mean conversion of Wan et al. [11] assumes an approximately normal underlying distribution and derives the standard deviation from the interquartile range. Because fewer than 10 trials were available, small-study effects and publication bias were not formally assessed, as such tests are unreliable with few studies. Analyses were conducted in Python 3.12 [13] (NumPy 2.4, SciPy 1.17, matplotlib 3.10); the analysis script is provided in Appendix C.

Results

Study Selection

**Figure 1:**
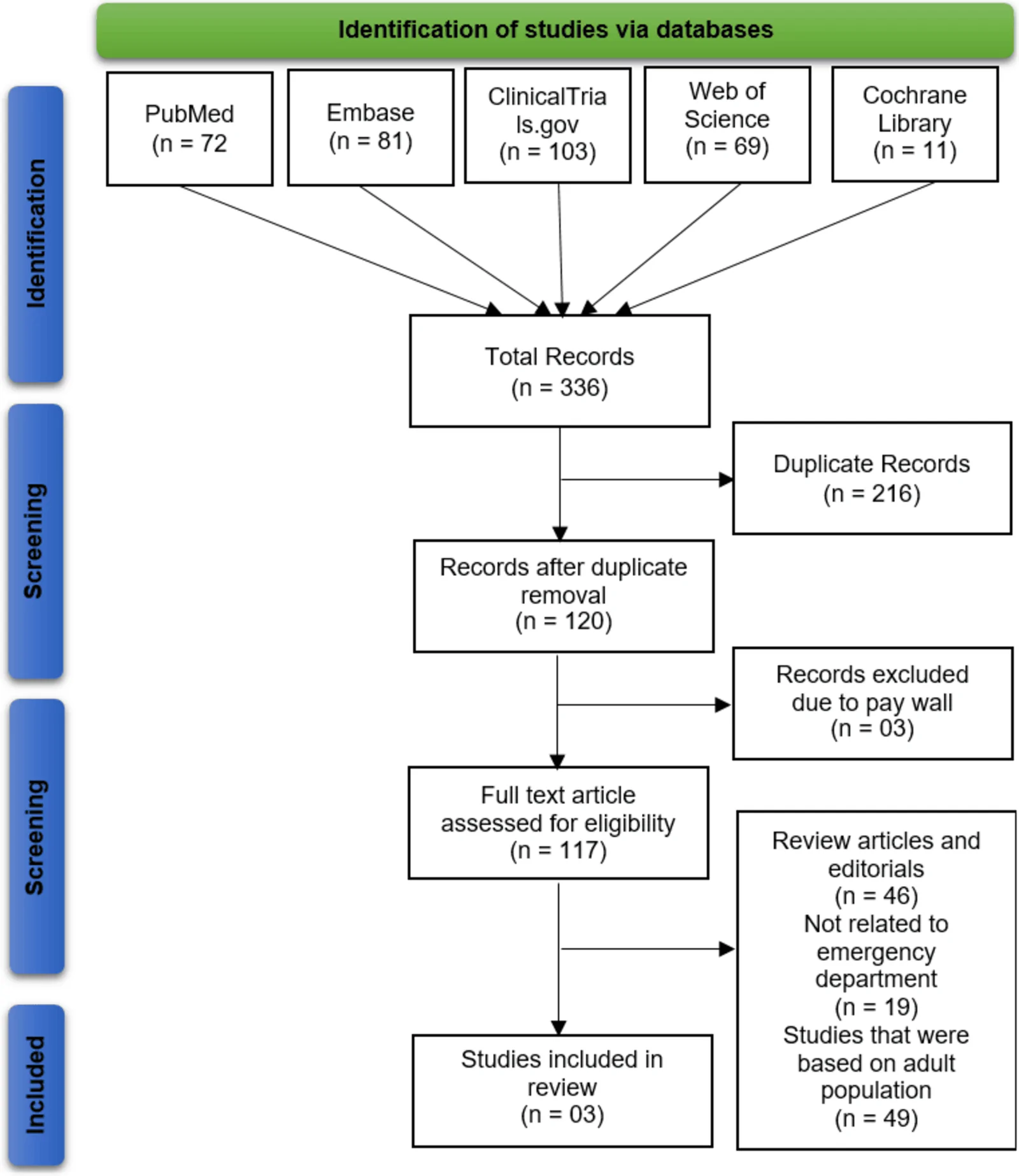
PRISMA 2020 flow diagram outlining the study selection process

The database search identified 336 records across five databases (PubMed, n = 72; Embase, n = 81; Cochrane Library, n = 11; Web of Science, n = 69; ClinicalTrials.gov, n = 103). After 216 duplicates were removed, 120 records were screened by title and abstract, of which 95 were excluded (46 reviews or editorials and 49 conducted in adult populations). Full texts were sought for the remaining 25 reports; three could not be retrieved because the full text was unavailable, leaving 22 reports assessed for eligibility. A further 19 were excluded as not related to emergency or acute presentation, leaving three randomized controlled trials that met all eligibility criteria [14-16]. These trials randomized 251 children (125 to adjunct systemic corticosteroids and 126 to control). The selection process is shown in Figure 1.

Characteristics of the Included Studies

The trials were clinically diverse (Table 2). Nagy et al. [14] studied a five-day course of intravenous methylprednisolone added to imipenem in 59 children (one to 12 years) with severe CAP. Tagarro et al. [15] (the multicenter CORTEEC trial) evaluated 48 hours of intravenous dexamethasone added to cefotaxime in 60 children (one month-14 years) with CAP and parapneumonic pleural effusion. Singh et al. [16] assessed intravenous dexamethasone for up to five days added to standard care in 132 children (two months-15 years) with WHO-defined severe CAP. The trials differed in corticosteroid agent, dose, and duration; in clinical phenotype; and in their primary endpoints (day-7 clinical/laboratory parameters, time to recovery, and treatment failure at 72 hours, respectively).

**Table 2:** Characteristics of the included trials RCT: randomized controlled trial; CAP: community-acquired pneumonia; mo: month; y: year; d: days; hsCRP: high-sensitivity C-reactive protein; q6h: every six hours; q12h: every 12 hours; h: hours

Trial	Country	Design	Population	Steroid regimen	n (steroid/control)	Primary outcome
Nagy et al., 2013 [14]	Hungary	Open-label RCT	Severe CAP, 1–12 y	Methylprednisolone 0.5–2.0 mg/kg IV, 5 d + imipenem	29 / 30	Day-7 fever, WBC, hsCRP
Tagarro et al., 2017 [15]	Spain	Double-blind RCT, multicentre	CAP + parapneumonic effusion, 1 mo–14 y	Dexamethasone 0.25 mg/kg IV q6h, 48 h + cefotaxime	30 / 30	Time to recovery (h)
Singh et al., 2026 [16]	India	Double-blind RCT	WHO-defined severe CAP, 2 mo–15 y	Dexamethasone 0.4 mg/kg IV q12h, up to 5 d + standard care	66 / 66	Treatment failure at 72 h

Risk of Bias

**Figure 2:**
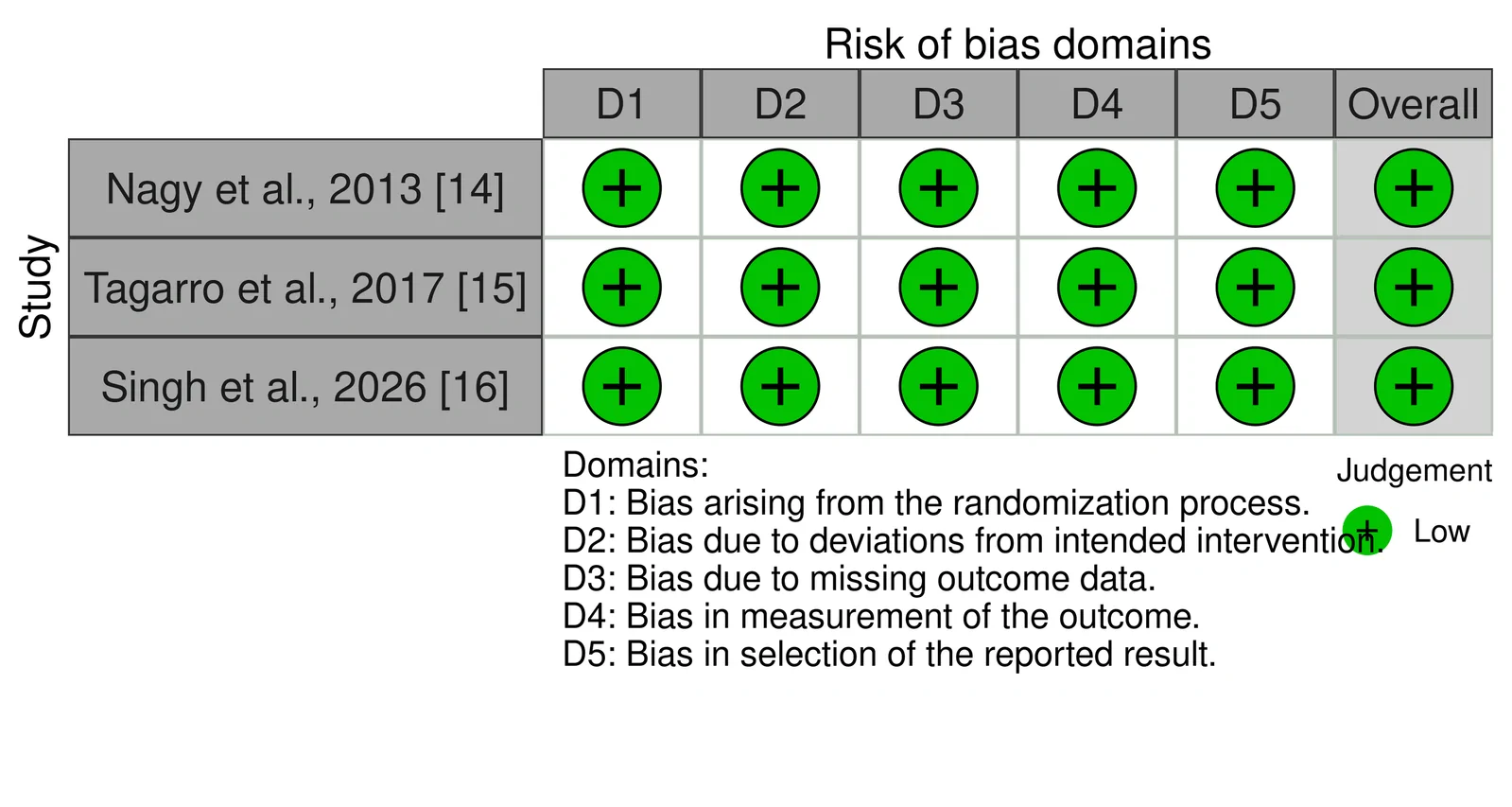
Risk-of-bias summary (Cochrane Risk of Bias 2 (RoB 2))

All three trials were judged at low risk of bias across all five domains and overall using the Cochrane RoB 2 tool (Figure 2). Randomization and allocation concealment were adequate, outcome data were complete with intention-to-treat analysis, outcomes were objectively defined and assessed (with independent verification where a trial was not blinded), and results were reported in line with the prespecified outcomes.

Length of Hospital Stay

**Figure 3:**
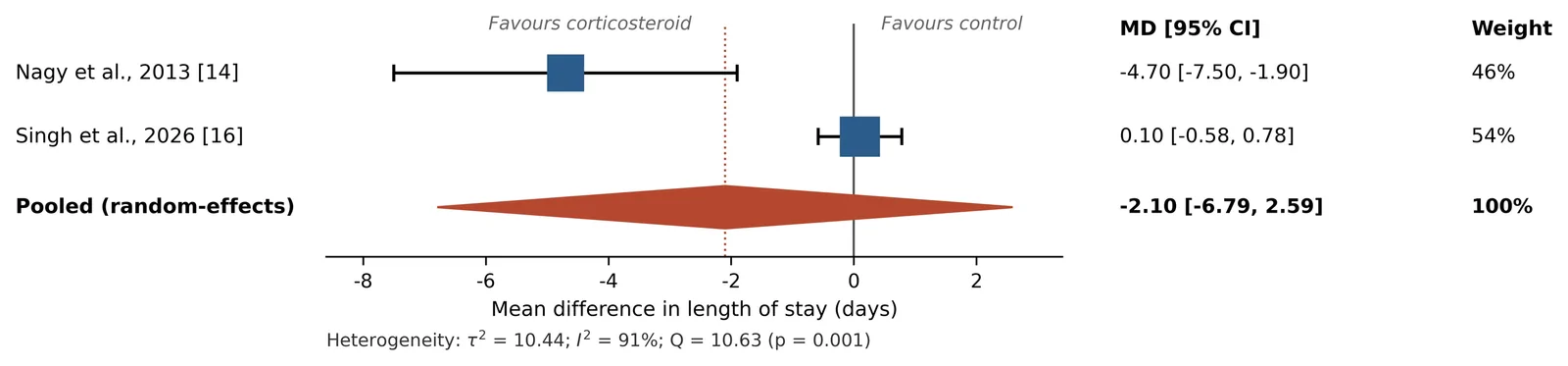
Random-effects meta-analysis of length of hospital stay (mean difference (MD), days). Negative values favor corticosteroids.

Nagy et al. [14] and Singh et al. [16] reported length of stay; Tagarro et al. [15] used time to recovery instead. The pooled mean difference favoured corticosteroids at −2.10 days (95% CI −6.79 to 2.59; p = 0.38), with substantial heterogeneity (I² = 91%; τ² = 10.4; Q = 10.6, p = 0.001) (Figure 3). This heterogeneity reflects the different clinical contexts of the two trials: Nagy et al. [14] reported a marked reduction with methylprednisolone (median 11.0 vs 16.5 days) where baseline hospitalizations were long, whereas Singh et al. [16], in a setting where the baseline stay was already short (median 6.0 vs 6.5 days), had little further room for reduction. The two trials are therefore most informative when read alongside their clinical context.

All-Cause Mortality

**Figure 4:**
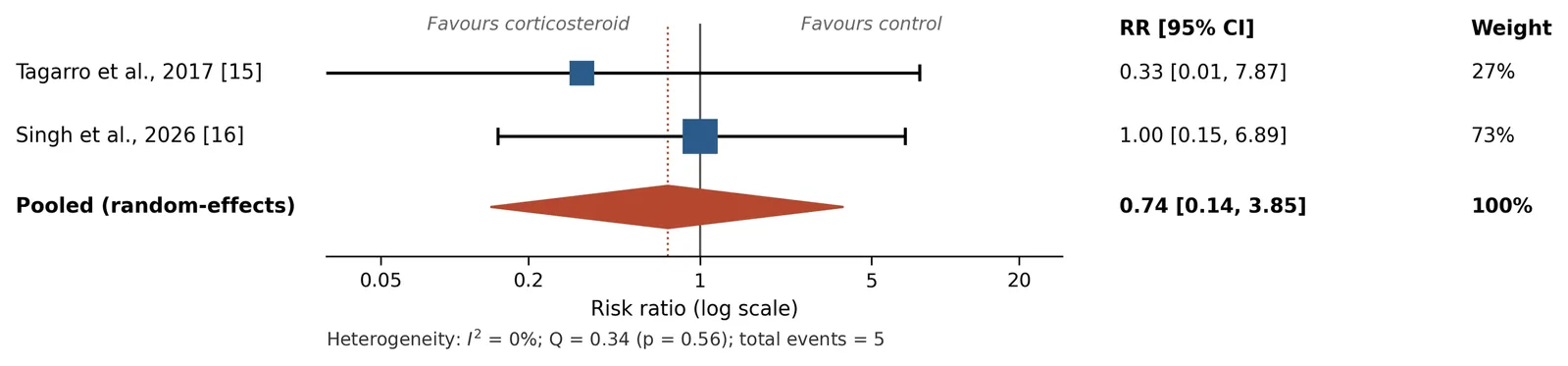
Random-effects meta-analysis of all-cause mortality (risk ratio (RR), log scale)

Mortality was reported by Tagarro et al. [15] (0/30 vs 1/30), and Singh et al. [16] (2/66 vs 2/66); Nagy et al. [14] did not report deaths. All-cause mortality did not differ between groups (pooled risk ratio 0.74, 95% CI 0.14 to 3.85; I² = 0%) (Figure 4), consistent with the low mortality expected in childhood CAP.

Trial-Specific Primary Outcomes

Because the trials measured different primary endpoints, these were synthesized narratively, and each trial demonstrated a significant benefit. Nagy et al. [14] found that adjunct methylprednisolone shortened fever duration (median 2.0 vs 4.0 days; p = 0.006) and lowered day-7 white-cell counts (8.4 vs 11.1 G/L; p = 0.023); the difference in high-sensitivity CRP (20.5 vs 35.1 mg/L) was not significant after adjustment (p = 0.53). Fewer corticosteroid-treated children developed complications (9/29 vs 18/30; p = 0.026) or required invasive procedures such as drainage or decortication (4 vs 16; p = 0.001). Tagarro et al. [15] reported that dexamethasone reduced the median time to recovery from 177 to 109 hours (~38%; p = 0.037; adjusted hazard ratio 1.95, 95% CI 1.10-3.45, p = 0.021); the benefit was concentrated in children with simple effusion (p = 0.017). Singh et al. [16] found that dexamethasone approximately halved treatment failure at 72 hours (25.8% vs 60.6%; hazard ratio 0.32, 95% CI 0.18-0.57; p = 0.001), driven by reductions in progression to respiratory failure (10.6% vs 31.8%; odds ratio 0.25; p = 0.004) and radiographic progression (22.7% vs 57.6%; odds ratio 0.22; p = 0.001).

Adverse Events

Adverse events were reported inconsistently and were not pooled. Tagarro et al. [15] observed more hyperglycaemia with dexamethasone (15/30 vs 6/30; risk ratio 2.5, 95% CI 1.2-5.5), mostly mild and transient, with a single child (later found to be prediabetic) requiring insulin. Singh et al. reported no significant excess of adverse events, and Nagy et al. [14] reported no treatment-related adverse events. Overall, corticosteroids were well tolerated, with transient hyperglycaemia the most consistent signal.

Discussion

This systematic review synthesized the randomized evidence on adjunct systemic corticosteroids in children with CAP and identified three eligible trials, together enrolling 251 children [14-16]. Each trial reported a statistically significant benefit on its own prespecified primary outcome: methylprednisolone shortened fever duration and reduced complications in severe CAP [14], dexamethasone shortened the time to recovery in CAP complicated by parapneumonic effusion [15], and dexamethasone approximately halved early treatment failure in severe CAP [16]. When outcomes reported by more than one trial were pooled, length of hospital stay favored corticosteroids, and mortality did not differ between groups. The consistency of benefit within each trial is noteworthy, but the pooled analyses are shaped by the clinical diversity of the trials and by the imprecision that follows from having so few studies, so the findings are best read as a consistent direction of effect rather than as a precise estimate of benefit.

The heterogeneity in length of stay (I² = 91%) is best understood as clinical rather than merely statistical. Nagy et al. studied severe CAP treated with intravenous methylprednisolone against a backdrop of long hospitalizations (median 11 to 16 days) in a single European center, whereas Singh et al. studied severe CAP treated with dexamethasone in an Indian setting where the baseline length of stay was less than half as long (median six to 6.5 days) [14,16]. When the baseline stay is already short, there is limited room for a large absolute reduction. The pooled point estimate was stable across DerSimonian-Laird, restricted maximum likelihood, and Paule-Mandel estimators, but the Hartung-Knapp adjustment produced a much wider interval, so the length-of-stay result should be regarded as directional rather than definitive and is best interpreted together with the clinical context of each trial.

Beyond baseline length of stay, the trials differed in ways that limit direct comparison and caution against treating them as evaluations of a single interchangeable therapy. The populations spanned wide and differing age ranges and distinct phenotypes; one trial enrolled children with parapneumonic pleural effusion while the others enrolled severe CAP, so the trials may represent conditions with different prognoses and treatment responses. The interventions also differed substantially in agent, dose, dosing interval, and duration, and the antibiotic backbone ranged from imipenem and cefotaxime to less standardized usual care. Expressed in prednisone-equivalent terms (relative glucocorticoid potencies of approximately 1.25 for methylprednisolone and 6.25 for dexamethasone), the methylprednisolone regimen delivered roughly 0.6-2.5 mg/kg/day of prednisone equivalent over five days, whereas the dexamethasone regimens delivered a substantially higher daily equivalent (approximately 5-6 mg/kg/day) over shorter courses of 48 hours to five days. These differences in potency-adjusted dose, duration, and co-treatment mean the trials cannot be regarded as testing a common class effect, and the pooled and narrative findings should be interpreted as the combined signal of related but non-identical interventions. Because age-stratified outcome data were not reported across the trials, a subgroup analysis by pediatric age band was not feasible.

The pediatric findings sit within a larger and more mature adult literature, and the comparison is instructive. In hospitalized adults, dexamethasone shortened the length of stay in a landmark double-blind trial [3], and adjunct prednisone shortened the time to clinical stability in a large multicenter trial, albeit with more hyperglycemia [4]. In severe CAP with a high inflammatory response, methylprednisolone reduced treatment failure [6], and the CAPE-COD trial demonstrated that early hydrocortisone reduced 28-day mortality among adults admitted to intensive care with severe CAP [7]. Contemporary meta-analyses of adult trials conclude that corticosteroids probably reduce the length of hospital and intensive care stays and, in the most severe presentations, mortality [5,17]. The pediatric signal for functional outcomes is directionally concordant with this adult evidence, while death is an exceedingly rare event in childhood CAP, so mortality is neither an achievable nor an appropriate primary target for pediatric trials.

A clinically important thread running through both the included trials and the wider literature is that benefit appears concentrated in inflammatory phenotypes. Tagarro et al. found that the reduction in time to recovery was significant among children with simple effusion [15], and Singh et al. observed that the treatment-failure benefit was driven by reductions in radiographic progression and progression to respiratory failure [16]. This mirrors the adult observation that the treatment-failure benefit of corticosteroids was demonstrated specifically in patients selected for a high C-reactive protein [6]. Taken together, these observations support a phenotype-directed approach in which the inflammatory milieu helps identify the children most likely to benefit, rather than the application of corticosteroids to every child with CAP.

The observational pediatric literature adds useful context. In a large multicenter analysis of children hospitalized with CAP, systemic corticosteroids were associated with a shorter length of stay, particularly among children who also received beta-agonists [8], and an emergency-department cohort examined child- and family-centered quality-of-life outcomes [18]. In the outpatient setting, corticosteroid co-prescription was not associated with reduced treatment failure [9]. These data suggest that the benefit is most evident in more severe or inflammation-driven presentations and the populations enrolled in the randomized trials and reinforce the value of selecting candidates by phenotype.

The safety profile across the three trials was reassuring. The most consistent adverse signal was transient hyperglycemia, reported at a higher frequency with dexamethasone by Tagarro et al. and generally mild [15], while Singh et al. and Nagy et al. reported no significant excess of adverse events [14,16]. This is congruent with adult experience, where hyperglycemia is the most reproducible and generally manageable harm [4]. Longer-term follow-up was not available in the included trials and would help characterize any delayed effects.

Several implications follow. Each trial demonstrated a functional benefit, and the therapy was well tolerated, which is encouraging; however, three small, clinically and therapeutically heterogeneous trials with imprecise pooled estimates cannot establish the size or generalizability of that benefit. The current evidence is therefore best regarded as preliminary and hypothesis-generating and does not support the routine use of adjunct corticosteroids in children with CAP. The consistent, favorable direction of effect, particularly in severe or inflammation-driven phenotypes, provides a strong rationale for an adequately powered, multicenter pediatric randomized trial using a single harmonized corticosteroid regimen, a prespecified inflammatory-enrichment strategy, a core outcome set that privileges patient-centered functional endpoints and length of stay, and follow-up long enough to characterize tolerability.

Limitations

This review has several limitations, most flowing from the size and diversity of the current evidence base. Only three randomized trials met the eligibility criteria, and they differed in corticosteroid agent, potency-adjusted dose, and duration; in antibiotic co-treatment; in clinical phenotype and age range; and in their primary outcomes, which limited the outcomes that could be pooled and precluded a common class-effect interpretation and age-stratified subgroup analysis. For length of stay, both contributing trials reported medians and interquartile ranges, which we converted to means and standard deviations using an established method [11] that assumes approximate normality; the pooled estimate was accompanied by substantial heterogeneity and, under the Hartung-Knapp adjustment, wide confidence limits, and therefore warrants cautious interpretation. The mortality analysis was based on few events because death is rare in childhood CAP, and one trial did not report deaths. The review was not prospectively registered, and three potentially relevant reports could not be retrieved; although these were not excluded on eligibility grounds, their unavailability is a limitation. Because fewer than 10 trials were available, small-study effects and publication bias could not be reliably assessed. Finally, no eligible trial recruited exclusively from the emergency department, so the evidence applies to the broader acute-care pediatric CAP population rather than to the ED decision point alone.

## Conclusions

Across three small, clinically heterogeneous randomized trials of children with CAP (251 participants in total), adjunct systemic corticosteroids improved each trial's own primary outcome: faster fever resolution and fewer complications, shorter time to recovery in parapneumonic effusion, and reduced early treatment failure in severe disease, and were well tolerated, with transient hyperglycemia the principal adverse effect. Pooled analyses favored corticosteroids for length of stay and showed no difference in mortality but were limited by the small number of trials, their clinical and therapeutic diversity, and the imprecision of the pooled estimates. The current evidence should therefore be regarded as preliminary and hypothesis-generating and is insufficient to support the routine use of adjunct corticosteroids in children with CAP. The consistent, favorable signal, particularly in severe or inflammation-driven phenotypes, provides a strong rationale for adequately powered, phenotype-enriched pediatric randomized trials with harmonized regimens and outcomes to determine whether, and in whom, adjunctive corticosteroids should be used.

## Appendices

Appendix 1

Search Strategy

Databases searched: PubMed, Embase, the Cochrane Library (CENTRAL), Web of Science, and ClinicalTrials.gov, from inception to the final search date. The final search date and the exact date each database was last searched should be recorded here. Example strategy (PubMed), adapted to the controlled vocabulary of each database:

("pneumonia"[MeSH Terms] OR "community-acquired pneumonia"[Title/Abstract] OR CAP[Title/Abstract]) AND ("adrenal cortex hormones"[MeSH Terms] OR corticosteroid*[Title/Abstract] OR glucocorticoid*[Title/Abstract] OR dexamethasone[Title/Abstract] OR methylprednisolone[Title/Abstract] OR prednisolone[Title/Abstract] OR prednisone[Title/Abstract] OR hydrocortisone[Title/Abstract]) AND ("child"[MeSH Terms] OR child*[Title/Abstract] OR paediatric*[Title/Abstract] OR pediatric*[Title/Abstract] OR infant*[Title/Abstract] OR adolescent*[Title/Abstract]) AND ("randomized controlled trial"[Publication Type] OR randomi*[Title/Abstract] OR placebo[Title/Abstract] OR trial[Title/Abstract])

Equivalent strategies using Emtree terms (Embase), the CENTRAL interface (Cochrane Library), topic searches (Web of Science), and condition/intervention fields (ClinicalTrials.gov) were applied.

Appendix B

Excluded Studies

1. Huang L, Gao X, Chen M. Early treatment with corticosteroids in patients with Mycoplasma pneumoniae pneumonia: a randomized clinical trial. J Trop Pediatr. 2014 Oct;60(5):338-42. doi: 10.1093/tropej/fmu022. Epub 2014 Apr 7. PMID: 24710342. 2. Bowles AK, Rodean J, Siegel EJ, Gray S, Vanja S, Howard LO, Orebiyi G, McDaniel CE, Mendoza JC, Edwards Y, McCulloch CE, Gonzales R, Auerbach AD, Kaiser SV. Examining Disparities in Pediatric Asthma, Pneumonia, and Bronchiolitis Care in General Hospitals. Hosp Pediatr. 2026 Jul 1;16(7):592-599. doi: 10.1542/hpeds.2025-008951. PMID: 42270087. 3. Yeh TF, Srinivasan G, Harris V, Pildes RS. Hydrocortisone therapy in meconium aspiration syndrome: a controlled study. J Pediatr. 1977 Jan;90(1):140-3. doi: 10.1016/S0022-3476(77)80789-0. PMID: 830880.

Appendix C

Statistical Reproducibility

Analyses were performed in Python 3.12 using NumPy 2.4, SciPy 1.17, and matplotlib 3.10. Pooled estimates used random-effects models with the DerSimonian-Laird between-study variance estimator; sensitivity analyses used restricted maximum likelihood and Paule-Mandel estimators and the Hartung-Knapp adjustment. Continuous outcomes reported as median (interquartile range) were converted to mean and standard deviation using the method of Wan et al., which assumes an approximately normal distribution and derives the standard deviation from the interquartile range. The complete analysis script is available from the authors on request.
